# Communication

**Published:** 1865-03

**Authors:** Geo. D. Winch

**Affiliations:** Surgeon 42nd Ill. Vols., Cairo, Illinois


### COMMUNICATION.

By GEO. D. WINCH, Surgeon 42d Ill. Vols., Cairo, Ill

Y——, aged 38, weak and anemic, was brought into hospital at 9 o'clock A. M., Nov. 17th, 1864, which was the first time I saw him.

He was seized with severe pain in the head and limbs, accompanied by a chill lasting nearly two hours, yesterday afternoon. I found him complaining of severe pain in the head and limbs, vomiting everything taken into the stomach, pulse only 100 and soft, extremities warm, pupils dilated They do not contract; neither does the patient suffer by exposure to light. The tongue coated white, and the coat very thick.

Nov. 18th.—Pulse continues same as yesterday; breathing laborious and noisy; vomiting ceased; he is quite stupid. At times he arouses and then complains of the pain in the head and limbs as being non-endurable.

Nov. 19th.—Is comatose; pupils largely dilated; pulse very weak and thread-like, not over 110; extremities warm. Expired at 2 A. M., Nov. 20th, without a convulsion, or any spasmodic actions, at least none were noticed.

Examined the brain at 10 A. M. the same morning he died. The following was its appearance: Spots of coaguable lymph the size of a ten cent piece lying near and on both sides of the longitudinal fissure. The membranes were agglutinated to the substance, but were easily separated from it. The prevailing character of the surface of these spots were gorged with dark blood, and there was a considerable quantity of serous fluid in the lateral ventricles.

